# Supplementary material for: Preliminary Study on the Formation Mechanism of Malformed Sweet Cherry (Prunus avium L.) Fruits in Southern China Using Transcriptome and Metabolome Data
Source: Int J Mol Sci. 2023 Dec 21;25(1):153. doi: 10.3390/ijms25010153 (PMC10779264; doi:10.3390/ijms25010153)
Supplement: Supplementary file 1 [file ijms-25-00153-s001.zip › supplementary figure.pdf]

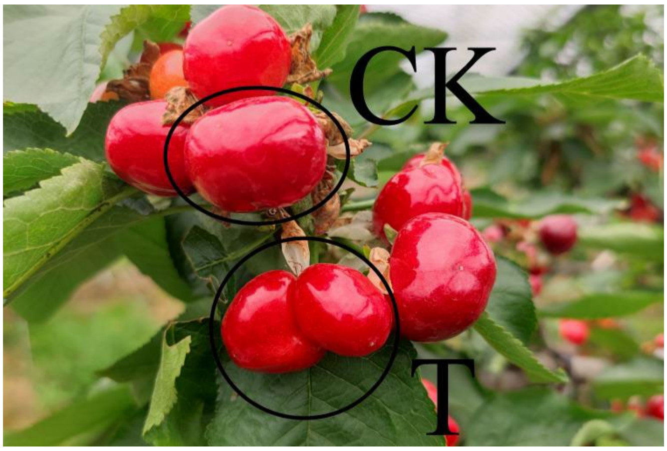

Figure S1. Normal fruit (CK) and Malformed fruit (T) of sweet cherry was selected as the experimental material.

Supplementary Figure 2

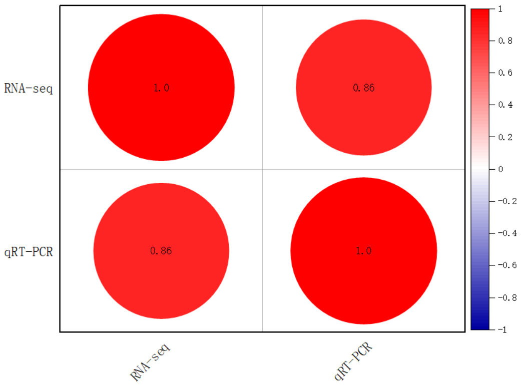

Figure S2. Correlation analysis of RNA-seq data and qRT-PCR in sweet cherry fruit.
