# Supplementary material for: Preliminary Study on the Formation Mechanism of Malformed Sweet Cherry (Prunus avium L.) Fruits in Southern China Using Transcriptome and Metabolome Data
Source: Int J Mol Sci. 2023 Dec 21;25(1):153. doi: 10.3390/ijms25010153 (PMC10779264; doi:10.3390/ijms25010153)
Supplement: Supplementary file 1 [file ijms-25-00153-s001.zip › supplementary table-revised.pdf]

Table S1. Transcriptome data of gibberellin-related enzymes

Gene Name	Gene ID	CK1	CK2	CK3	T1	T2	T3
GA20ox1	LOC110745003	1.58	1.97	2.66	5.44	4.01	5.84
GA20ox2	LOC110762932	1.65	2.06	1.51	4.27	4.16	5.28
GA20ox3	LOC110762934	3.54	3.14	2.83	4.92	7.42	5.22
GA20ox4	LOC110765963	4.31	4.67	4.71	7.13	10.25	8.96
GA20ox5	LOC110765965	28.71	49.51	37.59	56.94	46.45	61.13
GA2ox1	LOC110754555	2.13	3.41	2.77	4.69	4.79	4.89
GA2ox2	LOC110772934	18.79	8.72	10.69	46.20	46.69	59.32
GA2ox3	LOC110773997	5.01	6.25	3.12	0.12	0.75	0.40
GA3ox	LOC110750486	3.27	2.29	5.55	30.77	9.26	20.02

Table S2. Specific primers pairs for selected genes used in qRT-PCR.

Primers name	Sequences (5'-3')	Base (bp)
GA20ox1-F	CTCCATTCTTTGAAAGCCTCA	21
GA20ox1-R	GATCTTCACGATTTTCTTCGATAAT	25
GA20ox2-F	CCTCATTTCATAGCCTCTCCATT	23
GA20ox2-R	AAGCTCATCAGCGTGATCTTCAC	23
GA20ox3-F	AGGAGGTGTTTGATTTCGTAGC	22
GA20ox3-R	GGATTCTCAGGCCATTGGTT	20
GA20ox4-F	CCCTGATCCTGAGGACAAAG	20
GA20ox4-R	CAAGGCTATAAGTCCCATCAACT	23
GA20ox5-F	GGAGGAGCTACTGAGTGATGTAA	23
GA20ox5-R	GATGTTCTCCACCTCTTGTTTC	22
GA2ox1-F	GAACTGATGGCTGAAGGATTAA	22
GA2ox1-R	GCTCTGGACATGGTGGGTAG	20
GA2ox2-F	GTTCAAGAGCGTGAGGCATAG	21
GA2ox2-R	CCATAAGTGACGGCAGAGGT	20
GA2ox3-F	TCACAATCACAGCTCGTATTCA	22
GA2ox3-R	CAGTGGGTGGCTTTCGTCTA	20
GA3ox-F	AATGGGTGGAGGTGATGGTC	20
GA3ox-R	CAGCAAAGTTGAATGGCTTGTA	22
β-ACTIN-F	CCAGGGCTGTGTTTCCTTCTA	21
β-ACTIN-R	ATGATCTGCGTCATCTTTTCT	21
